# Abstracts &c.

**Published:** 1889-10

**Authors:** 


### ABSTRACTS AND GLEANINGS.

---

**Chloroform as a Routine Anæsthetic.**—How deeply men's minds are interested in the question, "Which is the safest anæsthetic?" has been amply shown by the prolonged correspondence which has followed our leading article dealing with the subject. We regret that pressure upon our space has prevented a wider discussion. At least two most opposed views are held by those who regard chloroform as a safe—nay, the safest—anæsthetic: holders of the one view contend that the less instructed the chloroformist is the better for the patient, provided he neglect the pulse, and watching the respiration, drag the tongue foward with artery forceps if any sign of respiratory trouble occurs; those who favor the other view, which has been advanced most recently by Mr. Foy of Dublin, assert that a large per centage of chloroform deaths are due to maladroit chloroformisation. But, as we pointed out, the danger of chloroform lies in the fact that a little carelessness on the part of the administrator will lead to fatal results. Ether when administered by itself has many disadvantages; but disadvantages are preferable to dangers, and so it seems desirable that it should oust chloroform as a routine anæsthetic, the latter agent being reserved for special operation and special individuals. When it is admitted, as we are glad to see it is, even by the strongest advocates of chloroform, that the utmost care is needed in its administration, our main contention is supported—viz., that were any casualty to occur under its influence, it would become the duty of the coroner to inquire whether due care had been exercised in the choice of the best anæsthetic for that individual case, and whether due care had been exercised in the choice of the best anæsthetic selected. It is not, of course, germane to the question to contend that many coroners and most coroner's juries are incompetent to settle such weighty questions. Their inefficiency is equally likely to be shown in the conduct of an inquest upon any case of poisoning.—*The Lancet.*

**Cocaine Poisoning.**—Richet and Langlois have recently made a series of experiments upon the toxic action of cocaine. They found, among other things, that animals could withstand a very much larger dose of the drug when placed in a cold bath, than when kept at or above ordinary temperature. This, therefore, may serve as a possible indication for treatment in cases of cocaine poisoning.—*Indiana Medical Journal.*

**Salicylates in Rheumatism.**—"In five hundred and thirty cases of acute rheumatism, collected by the British Medical Association, treatment with salicylate of sodium, it is well to know that a pleasanter and more efficient preparation can be secured by having the acid neutralized by sodium bicarbonate, than by using the commercial salt."—*Virginia Med. Monthly.*

The best way to give the drug is as follows: Dissolve the salicylic acid in equal parts of glycerine and mint water; dissolve nearly the same weight of sodium bicarbonate in a similar quantity of mint water. For each dose take equal quantities of each solution, pure or diluted, in cool water. Few patients will have difficulty in taking this, and none will object to it. If this is too much trouble, the following may be used. It has no peculiar therapeutic virtues, but experience has shown it to be an agreable mixture:

| | | |
|---|---|---|
| ℞ | Sodii Salicylat | dr. j. |
| | Glycerini | oz. ss. |
| | Aquæ menth. pip. | oz. jss. M. |

Better still is a similar solution of equal parts of salicylic acid and bicarbonate of sodium.—*Ind. Med. Jour.*

**Intubation.**—Prof. Jacobi (Post Graduate Clinic) presented a child, eighteen months old, which had been intubated seven weeks previously, and was still wearing the tube in its larynx. Intubation had been performed by Dr. Huber for ascending membranous croup. At the end of a week the tube was coughed up; no evil results were experienced at first, but at the end of twelve hours dyspnœa returned and grew quickly alarming. Replacing the tube gave immediate relief. At the end of the second week Dr. H. removed the tube, but was compelled to replace it within fifteen minutes on account of intense dyspnœa. Two weeks later it was again coughed up with a little blood and mucous. Dangerous symptoms supervened in a few hours. Since then the tube had been worn constantly, and at present was deeply imbedded in the larynx. The child is in good general health, experiences but little difficulty in nursing, the tube is manifest only by hissing respiration and cough. Dr. Jacobi regards the case as one of inflammation still present, with swelling following the removal of the tube; he excludes paralysis on account of the interval elapsing before the dyspnœa appears, and recommends non-interference for several weeks.

Prof. Jacobi treats laryngeal diphtheria by internal administration of bichloride of mercury.

| | | |
|---|---|---|
| ℞ | Hydrarg. chloridi corr. | gr. j. |
| | Aquæ | oz. viij. M. |

S. Teaspoonful every hour in a tablespoonful of water.

Add to this free use of stimulants, alimentation, and steam impregnated with turpentine vapors, and avoid sprays and local application by the mouth. Emetic of alum only if the membrane is partly detached. Intubation to be preferred to tracheotomy where practicable.—*Indiana Medical Journal.*

**Iodized Glycerin.**—"Dr. G. Hammond (London Med. Recorder) points out that a mixture of tincture of iodine and glycerin produces a greater effect on the skin than the pure tincture, possibly because the glycerin tends to prevent the evaporation of the iodine, and thus enables the whole of its powers to be utilized."—*Druggist's circular and Chemical Gazette.*

**Adherent Prepuce.**—In a case of adherent prepuce with elongation, Dr. Jacobi considered circumcision entirely unnecessary, recommending simply tearing up the adhesions and complete cleanliness.

**How Drunkards are Treated in Norway.**—The London correspondent of the *Am. Prac. and News* says that a well-known medical man, who has recently been in Norway, gives a glowing description of their manner of treating dipsomaniacs. A habitual drunkard in Sweden and Norway is treated as a criminal in this sense, that his inordinate love of strong drink renders him liable to imprisonment, and while in confinement it appears he is cured of his bad propensities on a plan that, though simple enough, is said to produce marvelous effects. From the day the confirmed drunkard is incarcerated no other food is served to him or her but bread and wine. The bread, however, it should be said, cannot be eaten apart from the wine, but is steeped in a bowl of it and left to soak thus an hour or more before the meal is served to the delinquent. The first day the habitual toper takes his food in this shape without the slightest repugnance; the second day he finds it less agreeable to his palate, and very quickly he evinces a positive aversion to it. Generally, the doctor states, eight or ten days of this regimen is more than sufficient to make a man loathe the sight of wine, and even refuse the prison dish set before him. This manner of curing drunkard habits is said to succeed almost without exception, and men or women who have undergone the treatment not only rarely return to their evil ways, but from sheer disgust they frequently become total abstainers afterward.—*Med. World.*

**A Novel Treatment of Tuberculosis.**—*Ed. N. Y. Med. Record*, May 4:—In the case of a disease so intractable as tuberculosis it is natural enough that many and diverse methods of treatment should have been proposed, since none of those who have gone before have been shown to exert any true specific action, although some have been of undoubted utility. One of the latest of these methods, and certainly one of the most startling in its originality, is that proposed, and actually carried out in three instances, by Dr. Lanigan, of Hyde Park, Mass. This method, which is described in the *Therapeutic Gazette* for February, 1889, consists in the attempt to substitute one cachexia for another. It is, of course, only a modification of bacterio-therapy, but its peculiarity lies in the manner in which the substitution is attempted.

Dr. Lanigan says that he has for many years held a belief in the antagonism between certain cachexias. Observing that there were inherited traits of character that were diametrically opposed by others, he set about ascertaining whether there were not also antagonistic inherited cachexias, the presence of one of which would preclude the existence of the other. He believes that he has discovered such antagonism in the case of phthisis and rheumatism, for of a considerable number of cases of the former disease, in which inquiries were made in this direction, he was unable to discover a single instance in which phthisis and rheumatism had ever attacked the same individual simultaneously or consecutively. He had also observed that rheumatics were always blessed with healthy lungs.

Thinking over this apparent antagonism of cachexias, Dr. Lanigan concluded to try the effect of turning a consumptive into a rheumatic. This was accomplished by transfusion of blood taken from a patient suffering from acute rheumatism. In one case he made two injections, each of four ounces, and in a second case a single injection of six ounces. In both instances the recipients of the blood were attacked with rheumatism within a week. In a third case, in which a single transfusion of eight ounces was made, there was a transient rise of temperature to 104° F., but no pain was experienced in the joints. All three patients improved, the temperature fell, the pulse became normal, and they increased in weight; and there is now every prospect, the author thinks, that they will continue to do well, for they are gaining strength, have a good appetite, and seem to be on the high road to recovery.

This is satisfactory as far as it goes, but it does not go very far. The report was prepared, apparently, but a short time after the experiments were made, and the assurance of continued improvement rests only upon the hopes of the operator. Dr. Lanigan, furthermore, says nothing about the physical signs, and but little beyond what we have quoted concerning the general symptoms. The author says, however, that he will answer any questions as to this method of treatment which may be asked of him, and those desiring information concerning these missing details may obtain it by addressing him.

[This theory of Dr. Lanigan strikes us as decidedly cranky.—ED. RECORD.]

**Anemone Pulsatilla in the Treatment of Uterine Affections.**—*Ed. Bost. Med. and Surg. Jour.*, May 23:—Charles Bovet, of Pogues, has made the above the subject of a paper before the *Societe de Medecine Pratique.*

After alluding to the benefits of pulsatilla in blennorrhagic orchitis, relieving the pain and dispersing the swelling in a very few days, he affirmed the same remedy to be of singular efficacy in certain painful uterine affections. Thus, the pains which accompany difficult menstruation are often speedily mitigated by the pulsatilla; also those which result from metritis, ovaritis, salpigitis.

Bovet thinks that pulsatilla has an action of the nervous system similar to that of aconitina of certain neuralgias; this action becomes elective in manifesting itself particularly on the nervous filiments of the uterine apparatus.

The tincture made from the dried plant is, he says, a poor preparation; nothing is trustworthy but the alcoholic tincture, made by macerating the fresh leaves in strong alcohol. The plant should be gathered for this purpose about the middle of June.

The directions for the administration of the medicament are as follows: Five days before the expected menstrual period, give ten drops of the tincture four times a day in a tablespoonful of wine; suppress the medicine during the three or four days of the menstrual flux, then resume it in the same doses during the five days which follow. In cases of ovarialgia, caused by a chronic phlegmasia of the uterus or its annexes, the medicine is continued in the same doses until the pain disappears.—*Epit.*

**Opium Poisoning in an Infant.**—By J. Henry Heavrin, M. D., Curdsville, Ky.—On the morning of June 12, 1888, I was summoned to attend the child of James C———, living two miles from the city. On my arrival an hour later I found that the mother had given her three-year-old daughter ʒ j of tinct. opii. deod. through a mistake. I saw that I had a very bad case of opium poisoning to deal with. The opium had been taken about two hours before I saw the child, and she was now very drowsy and could only be aroused with difficulty.

I at once ordered fifteen grains of zinc sulph. in a wineglass of warm water, but, owing to the age and drowsiness of the child, I could only succeed in getting about one-fourth of it down her, which failed to produce emesis. I then ordered a glass of warm mustard water and repeated the zinc sulph. About twenty minutes afterwards the child vomited slightly twice, throwing up mostly all the breakfast she had eaten that morning. I then ordered, at 10 A. M., a strong cup of coffee, and to keep the child amused and roused up. At 11 A. M. the respiration was twelve per minute. I repeated the strong coffee and gave a hypodermic injection of atropia sulph. At 12 noon her respiration was eight per minute.

I at once gave her a hypodermic injection of strych. sulph. At 12:30 P. M. the child's breathing was only four per minute. At 1 P. M. I repeated the strych. sulph., gave her a little black coffee, and applied the fringes of a towel wrung out of ice-water to her chest and back. At 1:30 P. M. her respiration was still four per minute. I still kept up treatment, repeating the black coffee and using the iced towel, and also kept her in motion all the time. At 2 P. M. her respiration was three per minute. As I had seen no improvement from the strychnia, I left it off and repeated the atropia sulph. At 3 P. M. her respiration was four per minute, having raised one in the last half hour.

The breathing kept on improving slowly, and at 6 P. M. I left. The respiration was then eight per minute. Directed them not to let patient go to sleep and to still use the coffee and the wet pack.

At 12 midnight I saw the patient again. Respiration by this time had raised to twelve per minute and was fairly good; she could be easily roused. I directed them to undress her and put her to bed, waking her at intervals. Next morning at 9 o'clock she was still sleepy, but had taken some breakfast and was in fairly good health.

The following points were observed:

1. This was the youngest patient to have taken as much opium and recover I ever saw.

2. I do not think the strych. sulph. proved to be of any value in my hands.

3. I prefer the ice towel, black coffee, hypodermic injections of atropia sulph., and patient kept awake by some means, to any treatment I have ever tried.—*Epit.*

**Treatment of Eczema in Children.**—According to Beenier, in the *La Pratique Medicale*, eczema of the face in children may be recognized in three different forms. The first is met with in lymphatic or scrofulo-tubercular subjects, is but slightly pruriginous, develops around the orifices of the nose and mouth, and often causes glandular complications, which may be the starting point of tubercular lesions. Its secretion is very abundant, and is frequently accompanied by phlyctenular keratitis.

The second form of eczema covers the entire face like a mask, and sometimes exists also on the back of the hands, accompanied by buccal irritation, such as sensitiveness of the gums and abundant salivation, indicating the close connection that this affection has with dentition; consequently the best means of combating this form is to diminish the irritation of the gums. This may be accomplished by rubbing the gums with the following preparation: ℞. Glycerin, min. clx; Distilled water, min. clx; Bromide of potassium, gr. xv; Cocaine, gr. viiss. If the sleep is at all disturbed, bromide of potassium may be given internally, while an ointment of oxide of zinc and vaseline may be employed externally.

The third form of eczema differs from the preceding in the absence of keratitis, and in the fact that it commences usually on the scalp, where it is characterized by an abundant desquamation, and may then extend to the eyebrows, the face, and even the neck, back and shoulders. Its mode of treatment is very simple; the hair should be cut and the surface well washed with soap, and the face should be washed with warm water, to which a little milk may be added to remove all the oils, and then an ointment applied externally in the following proportions: ℞. Resorcin, 1 part; Oxide of zinc, 10 parts; Vaseline, 100 parts. Or the oxide of zinc may be replaced by sulphur in quantity of sixty to seventy-five grains. In the case of scrofulo-tubercular eczema, the gravest form of all, the surface should be made of ointment of calomel, one part to thirty of vaseline. But as this preparation, as well as the resorcin prescriptions which may be used, may be irritating, it must be carefully watched.—*Therapeutic Gazette.*

**Herpes Circinatus.**—I have met many of these cases which were thought very difficult to treat. I have seen large patches on different parts of the body which were constantly spreading, more especially on the face and arms. I have also found them on the scrotum and penis of the male and upon the vulva of the female, producing irritation, and spreading. I have seen various kinds of treatment used, some of which did not prove of any avail. The best treatment that I have met with is the painting of the parts with the muriated tincture of iron, using it full strength. Two or three applications with a camel's hair brush will stop the disease every time. I have also used the same application for herpes zoster and cured it. It generally takes more applications. This is no guess work, but I have tried it in over one hundred cases in the last fifteen years. If my professional brethren who have trouble in treating these cases will try this they will not be disappointed and have their patients go elsewhere for treatment.—DR. WM. HENRY, in *St. Louis Med. Journal.*

**What Semen has done for Brown-Sequard.**—By the subcutaneous injection of semen obtained from a dog, Brown-Sequard claims to have restored the vigor of his young manhood. He took the testicles of dogs just castrated; by compression and mashing he extracted from these glands a liquid which he subcutaneously injected in himself. In speaking of the physiological effects of these injections, he says: "All that I have no longer been able to do, or that I have done poorly for many years past by reason of my great age, I am able to do to-day very effectively. I was affected with an obstinate constipation, due to paresis of the colon and rectum; now my bowels are perfectly regular, without the necessity of any laxative; I urinate passably well, and the projective force of my jet of urine has tripled, which proves that my bladder has recovered its former vigor. I have had a similar experience with regard to my other organs, as well as my limbs. I can remain standing for three hours without feeling the least sense of fatigue. But this is not all; I am in a better condition for work than ever. For a long time it was impossible for me to apply myself to any brain work in the afternoon; now I can indulge in my ordinary tasks without difficulty, consequently there is both an increase of physical and mental vigor, which I owe to these injections. I am really thirty years younger thereby."—*Alabama Medical and Surgical Age.*

**Antipyrin Epilepsy.**—All cases of poisoning by this drug are important, since, from its undoubted utility not only in fever, but also in neuralgias, it has become very popular. Tuczek has recorded a case in a boy æt. 9 years, to whom the drug was given to allay the paroxysms of whooping-cough. The patient had never suffered from convulsions, rickets, or worms. The dose given was about 17 grains daily in three doses for the space of three weeks. At the end of this period the patient was seized with vomiting, and passed into a state of somnolence, ending in deep sleep. Rapid ensuing epileptiform spasms followed, sometimes general, sometimes unilateral, accompanied with grinding of the teeth and jactitation, arythmia of the cardiac beat, and dilatation of the pupils. A macular eruption appeared on the skin, and the temperature became subnormal, while the pulse was slow and tense. On the third day of poisoning, consciousness began to return, the convulsions diminished in severity, and ceased entirely on the fourth day. For a few days the child was depressed, but completely recovered. It is interesting to find that during the whole period of poisoning there was acetonuria—a condition which is ascribed by Tuczek to the increased destruction of the albuminoid constituents of the body caused by antipyrin. During the poisoning there were, as might be expected, no attacks of whooping-cough, but afterwards the paroxysms returned with increased severity, and lasted for some months.—*Brit. Med. Jour.*, *W. M. A.*

**A Cure of Nocturnal Incontinence.**—Dr. R. Jamin reports ("Abeille Medicale," May 13, 1889) favorable results from the electrical treatment of a case of nocturnal incontinence of urine in the case of a young girl aged fifteen, in whom he and others had obtained no amelioration by prolonged medicinal treatment. He employed direct faradization with an olive-tipped bougie applied to the urethra, after the advice of Guyon. The girl had urinated in bed nearly every night for a number of years. After applications she only micturated in bed once during a period of four months.—*N. Y. Med. Jour.*

**Note on some Gynecic uses of Boric Acid.**—This was the title of Dr. Potter's paper read (*American Journal of Obstetrics*) at the Newport Session of the Association.

A *resume* of personal experiences with the drug was given, in which it was ascertained by the author that boric acid is a most excellent substitute for iodoform for many purposes for which the latter drug is frequently employed. It is chemically suited to neutralize the acrid secretions of the uterus and vagina that irritated the genital tract, and which sometimes cause sterility by destroying the fecundating power of the spermatozoa.

Through its free use in the vagina, it contributes to the better management of uterine and ovarian displacements by vaginal tamponnement, permitting the retention of the tampon for a week or more without putrescence; for similar reasons it makes the V. tamponnement a more potent agent in treatment of pelvic inflammatory residues. Through its agency the manipulations of the genital tract become less frequently necessary, because of the more lasting antiseptic properties of this drug, and because of the odorless, and non-irritating qualities.

After plastic operations in the genital tract, it may be used freely in the vagina, and with boric cotton furnishes a suitable antiseptic dressing to guard the lines of coaptation from the secretions that they may interfere with perfect primary union.—*Ala. Med. Jonrnal.*

**Dr. W. M. Beek writes** to *Medical Record*, July 27, 1889, that in the early stages of Quinzy he has found chloral hydrate nearly a specific, three or four grains to the ounce of glycerine being used as a gargle. Is efficiency and *modus operandi* are at once apparent when we consider that it is locally antiseptic, astringent and sedative.

**Leprosy and Syphilis.**—No malady has a more curious history than leprosy, and nothing in that history is of greater interest than its possible relations to syphilis. Even in modern times there have not been wanting men of weight who have upheld with Simon the view that the venereal disease is in some way derived from the older malady. For the following facts still remain unexplained.

Throughout the middle ages, until the end of the 14th century, and especially during the crusades, leprosy was endemic throughout the civilized world. No country escaped its ravages, and historians tell us that in England alone, with perhaps one-tenth of its present population, there were over five hundred houses devoted to the care of sufferers from this malady, and called leproseries or lazar houses. Numerous religious orders, male and female, devoted themselves to the care of the unfortunate sufferers. It was universally believed to be contagious, and the leper was avoided by society and forcibly segregated. There is no doubt that this chief scourge of Mediæval Europe, which caused hundreds of thousands of deaths, was the same malady as that which we now call leprosy.

With the beginning of the 15th century leprosy began to abate, the notices of it in contemporary writers became fewer and fewer; the lazar houses emptied and gradually disappeared. As mysteriously as it arose leprosy vanished from amongst civilized society. So completely did it disappear that the disease itself became a myth, and was practically rediscovered in Scandinavia in the forties by Boeck and Danielssen.

But a new and even more formidable malady arose in its place. First noticed among the soldiers in the hostile camps at Naples; the French called it "*le mal de Naples*," and the Italians called it "*le mal Francaise*." Finally the onus was laid upon shoulders which were unable to repudiate the burden, and this new plague was supposed to have been brought upon suffering humanity from America by the sailors of Columbus.

Syphilis spread like wild-fire throughout Europe in the 16th century. "From the Emperor on his throne," says a contemporary writer, "to the lowest civilian in Christendom, none is exempt." The low state of morality then prevalent, together with the unhygienic and dirty habits that were universal, sufficiently explain its spread. The soil was a virgin one, and the malady raged with a virulence and a universality utterly foreign to it to-day. Gradually it wore itself out and became the comparatively mild affection that it now is.

Meantime leprosy had apparently vanished from the face of the earth. But when in the latter part of the 18th and the first half of the 19th century syphilis had begun to abate, leprosy reappeared.

First found in obscure corners of Europe and Asia, it has gradually spread abroad again. It is spreading now as surely as syphilis is abating in syphilized population. Whereas, twenty years ago, Hebra could specify individual cases of Lepra observed in this or that country by this or that observer, it is safe to assert that there is not a great city in the world to-day that does not contain its cases of leprosy. Is it possible that a malady that almost perished from inanition in an exhausted soil may start afresh when that soil has lain fallow, or has had other crops for several generations?

It does not seem probable that there is any etiological relationship between the diseases. But the history of their occurrence and prevalence forms a chapter of rare interest to the student of disease, and a promising field for future investigators.—*Int. Jour. of Surgery.*

**A New Method of Illumination of the Larynx.**—Professor Voltolini has, for a period extending over a number of years back, made experiments in endo-laryngoscopy by a method which he was the first to put in practice. This method consists in the illumination of the larynx from the outside, profiting by the natural transparency of the laryngeal and tracheal walls.

Czermak was the first to mention the possibility of this procedure and called attention to the brilliant reddish tinge acquired by the glottis when a strong ray of sunlight was thrown upon the exterior of the throat.

The obvious disadvantages of sunlight, however, which hitherto had alone been used, caused the abandonment of the procedure by the first generation of laryngologists, and little reference is made to it in modern text books on the subject.

Professor Voltolini's modification of the method removes these objections and enables us to put it to practical use in laryngeal examinations. He employs for illuminating purposes an electric lamp provided with a reflector, whose light is thrown directly against the patient's throat. Then, by means of the usual mirror, a perfect inspection of the larynx is possible. The best results are obtained in persons with thin necks, as well as in children whose delicate tissues are easily permeated by the light.

Professor Voltolini cites a number of cases in which his method gave desirable results, and we are of the opinion that it may prove a valuable adjuvant to the common manner of examination that has been in vogue since the days of Czermak.—*Ib.*

**Use of the Testicles as Reinvigorators.**—Dr. McCormac, of Mansfield, Oregon, writes as follows to the Medical News:

For over twenty years, to the personal knowledge of the writer, and probably for as many centuries, the Chinese have used the testicles of the sea lion for the purpose of stimulating the organs of generation in man and as a general nerve stimulant.

A great many sea lions are killed in the immediate vicinity of this place, principally for their oil; but the bristles and penis, with testicles, are sold to agents of the Chinese. The first named are used for ornaments, but the last two articles are used by them as medicine. At times âs many as one hundred and fifty are seen drying in the sun. When needed, a portion of a testicle is grated into a vessel, and an infusion is made which is administered *Pro re nata.*

**Treatment of Ganglions.**—Ganglion is the name given to an enlarged bursa which is developed in connection with one of the tendons, being most common on the back of the hand, or on the extensor tendons of the thumb. It forms a little hard swelling on the back of the joint, and often causes a degree of weakness of the hand which seems out of all proportion with the seeming triviality of the affection.

In olden times the treatment of ganglionic swellings was to give it a smart blow with a book or other body. We adopt in a great preference to this course and old-fashioned treatment which was not only less certain and more painful but unnecessarily rough and unsurgical, the following, which rarely fails to obtain an early, if not an immediate cure. Its object is to evacuate the entire contents of the cyst, and to bring its opposite surfaces into perfect opposition with each other. It is a small operation; but on the delicacy of its performance its success materially depends. Bending the hand foward, in order to tighten the skin over the cyst we would pass vertically into the center of the tumor a broad shouldered lancet. By a lateral movement of the instrument the orifice will be dilated, and the contents will freely escape. Now it is indispensable to the obliteration of the cyst that the whole of its contents should be evacuated—every drop and every fraction of a drop, to effect which the sac must be compressed and kneaded in every direction. We therefore then apply a well made, thick compress of lint, and strap it down tightly with good plasters, and lastly apply a roller. In forty-eight hours the wound is healed, and the ganglion is seen no more. We are led to allude to this subject, by the fact that during the last six months we have seen a dozen or more of these little bodies—more than we had before seen in as many years.—*Mass. Medical Journal.*

**Brown-Sequard Injection** is prepared as follows: The testicles of dogs or guinea-pigs are cleansed of their capsules, three or four times their volume of distilled water is added, they are thoroughly triturated in a mortar and filtered. When filtered through a paper filter the liquid is of a reddish hue and opaque. It is almost clear and transparent when a Pastgur's filter was employed. The latter liquid is followed by less pain and other bad effects. A cubic centimeter is sufficient for a single injection.—*Ex.*

**Treatment of Otorrhœa.**—Dr. Robert L. Randolph, of Baltimore, sums up an article in the *Medical Record* by saying that, 1. Carbolic acid, to get its full antiseptic value, must be employed in a solution too strong to be used in the ear without doing positive harm. 2. Iodoform will not be tolerated by the majority of patients, and did give me as good results as boric acid. 3. Inasmuch as the recent experience of aural surgeons has shown that the use of boric acid in the powder form is associated with danger, I comparatively seldom use the agent in this form. In the saturated solution boric acid is a valuable agent. 4. If the ear be thoroughly cleansed with warm water, and the acid sublimate solution be allowed to remain in for ten minutes or more, and the ear then closed with a pledget of cotton which has been moistened with the solution, the clinical observations of the past few months have led me to conclude that this solution has a somewhat larger percentage of cures than the other three agents.

The following is the prescription used:

| | | |
|---|---|---|
| ℞ | Hydrarg. bichlor. | gr. ss. |
| | Acid. tartar | grs. xx. |
| | Aquæ | q. s. ad ℥ v. |

The patient is first required to syringe the ear out with warm water, and then to pour the sublimate solution into the ear till the latter is quite full. The fluid is allowed to run out after remaining in the ear ten or fifteen minutes. A piece of cotton is then moistened with the solution, and with it the external opening of the ear is closed. This treatment is repeated two or three times a day. As far as possible, then, the tissues of the drum-cavity, its remote connections, and the whole external auditory canal are kept in a condition unfavorable for the growth of organisms. A marked diminution in the discharge is seen almost immediately, and not infrequently a patient will remark upon the absence of oder after the first day's applications.—*Practice.*

**Sunflower in Malarial Fevers.**—In the *Meditzina*, No. 21. 1889' p. 7, Dr. S. Kazatchkoff emphatically draws attention to the common sunflower (*Heleanthus annuus*, Russ. *podsolnetchnik*) as an excellent and cheap substitute for quinine in the treatment of malarial fevers of all possible forms. The remedy has been from time immemorial used for the purpose in the Russian, as well as Persian and Turkish popular medicine, and that mostly after the following plan: A flask is filled up loosely with finely-cut dry or recent flowers and stem, and then with *vodka* (aqua vitæ). The (hermetically corked) vessel is left to stand under sun rays, or at some warm place, for two or three days. The tincture is then ready for use and should be given as a small wineglassful (a *liqueur* glassful) three times a day. In recent cases, complete and permanent cure ensues in from one to three days, in most obstinate and inveterate cases not later than a week. The remedy proved successful even in such cases where quinine and other anti-malarial means failed. About ten years ago the sunflower was similarly recommended (*Meditzinskoie Obozrenie*, September, 1879) by Dr. Petr Filatoff, of Saransk.—*St. Louis Med. Jour.*

**Treatment of Carbuncles.**—The method of crucial incisions has long been a favorite method of treating carbuncles, and certainly, when thoroughly done, greatly abbreviates the duration of the malady. Some time ago I remember to have read in some medical journal, a recommendation not to poultice a carbuncle when opened, but to apply a large sponge wet in some disinfectant solution, carbolic acid or corrosive sublimate. This is a very sensible procedure, as I can testify from experience. The sponge should be large enough to completely cover the carbuncle and may be cut into shape so as to fit over it like a cap. Before being applied, it is dipped into a sublimate solution, 1 part to 2000, or a two per cent carbolic solution; a little iodoform may then be dusted into the cavity of the carbuncle, down to the bottom of the incision, and the sponge is then adjusted by a few turns of a roller bandage. There is no need of poulticing, for pain and tension are removed by the incisions; the microbes are more effectually stopped in their destructive depredations by the antiseptic liquid which is thus enabled to penetrate every part, than they can be by any other method; the dead shreds of tissue will rapidly separate under the disinfectant dressing, and all the discharges will soak into the sponge and be kept from putrefaction. Night and morning the dressings are renewed; the sponge, full of purulent matter and debris, is thrown into a bucket of boiling water, and afterwards cleansed and again soaked in the sublimate solution for a fresh application. Simultaneous with the separation of sloughs, granulations make their appearance, and *restitutio ad integrum* rapidly takes place.—*Medical Age.*

**Grafts of Frog Skin in Chronic Ulcers.**—In the *Russkaia Meditzina*, Dr. Nesteroosky relates four cases of old-standing, intractable extensive and deep ulcers of the leg, foot,and thigh, where, after all ordinary means had failed, the transplantation of grafts of frog's skin was invariably followed by a permanent healing in from nine to fourteen days. Dr. Nesteroosky takes an ordinary water frog and keeps the lower portion of its body immersed in a sublimate solution (1 to 1,000) for five minutes: then he pinches up a piece of skin on the abodomen with forceps, and cuts out as many grafts as are required, each the size of a finger nail. Having washed the pices as well as the ulcer with a four per cent. solution of boracic acid, he carefully places the grafts on the granulating surface, and covers the part with a layer of boracic gauze and a piece of tow, fixing the whole with wax cloth and a starched gauze roller. The dressing is changed and the ulcer washed first on the third or fifth day. The writer summarizes his experience as follows: 1. In all cases of extensive and badly cicatrizing ulcers, skin grafting is indicated. 2. Skin which is quite free from glands and hairs is most suitable for the purpose. 3. Frog's skin completely satisfies those conditions. 4. The method is simple, safe, easily used everywhere, cheap, and most effective.—*British Med. Journal*, June 1, 1889.

**More Experience with the "Elixir of Life."**—S. C. Showalter, of Dayton, O., æt. 60, voluntarily submitted to injection of elixir of life three weeks ago, hoping for relief from rheumatism, and died on the 2d inst. from the effects of the treatment. Immmediately after the injection was made his limbs began to swell and his whole system was permeated with blood poison.—*The Journal.*

